# Validation of the SCOPETAS Scale for Nursing Professionals in Pediatric Interhospital Transport

**DOI:** 10.3390/nursrep16020067

**Published:** 2026-02-14

**Authors:** Marina Medina-Valles, Ana Elisa Laso-Alonso, Alberto Medina-Villanueva, Vicent Modesto-i-Alapont, David Zuazua Rico, Alba Maestro-Gonzalez

**Affiliations:** 1 Hospital Universitario Central de Asturias, 33011 Oviedo, Spain; marinamedinavalles@gmail.com (M.M.-V.); amedinavillanueva@gmail.com (A.M.-V.); 2 Hospital Vital Álvarez Buylla, 33619 Mieres, Spain; anaelisalasoalonso@gmail.com; 3 Hospital Universitario y Politécnico La Fe, 46026 Valencia, Spain; vicent.modesto@gmail.com; 4 Faculty of Medicine and Health Science, University of Oviedo, 33006 Oviedo, Spain; maestroalba@uniovi.es

**Keywords:** pediatric nursing, interhospital transport, triage, scale validation, clinical decision-making, nursing autonomy

## Abstract

**Background**: Pediatric Interhospital Transport demands highly specialized, coordinated care to ensure safety and continuity across settings. The SCOPETAS scale, recently adapted into Spanish from the Pediatric Transport Triage Tool, has been validated for physicians but not for nurses. **Objective**: To validate the SCOPETAS scale for use by nursing professionals in Pediatric Interhospital Transport. **Methods**: A cross-sectional inter-rater reliability study using clinical vignettes was conducted between December 2024 and February 2025 with nurses from eight hospitals within a Spanish autonomous community. Participants applied the SCOPETAS scale to two simulated pediatric transport scenarios. Agreement with physicians’ decisions (gold standard) was analyzed using weighted kappa statistics, logistic regression, and ROC curve analysis. **Results**: A total of 128 nurses participated (91% female; mean age, 39.5 years). Correct team composition decisions were achieved in 91.4% of severe cases and 73.9% of mild cases. Overall concordance with physicians was high. Possession of a Master’s degree was inversely associated with decision accuracy. **Conclusions**: When applied by nurses, the SCOPETAS scale demonstrated strong agreement with physicians’ decisions, particularly in severe scenarios. Its implementation may enhance patient safety, optimize resource allocation, and promote nursing autonomy in Pediatric Interhospital Transport, supporting its integration into clinical practice as a validated, evidence-based decision-support tool for pediatric transport triage.

## 1. Introduction

Interhospital transport (IHT) is a vital component of healthcare delivery, especially when patients require urgent or specialized care unavailable at the referring facility [1]. Unlike primary emergency transport, which prioritizes rapid transfer to the nearest hospital, IHT aims to ensure continuity and quality of care by relocating patients to centers equipped to provide the necessary specialized treatment. In pediatric populations, interhospital transfer of critically ill patients carries a significant risk of adverse events, especially when performed by non-specialized teams, with historical rates ranging from 5% to 20% [2,3]. Furthermore, it promotes equity in access to high-complexity services, particularly for patients from rural or peripheral areas.

Despite its importance, there is no internationally standardized model for pediatric transport teams, resulting in considerable variability in resources, competencies, and clinical practice. In Spain, the *Sistema de Valoración de Pacientes para el Transporte Secundario* (SVPTS) provides guidance for adjusting team composition according to patient severity in adult critical care, but its applicability is limited by regional heterogeneity and its adult-oriented design [4].

In pediatrics, validated tools are scarce. Two scales have been developed: a pediatric adaptation of SVPTS and the Pediatric Transport Triage Tool (PT3) [5], based on the Pediatric Early Warning Score (PEWS) [6]. PT3 incorporates critical transport-related factors such as sedation, vasoactive drugs, and advanced respiratory support. Recently, the PT3 tool underwent cultural adaptation into Spanish and was validated among physicians, resulting in the SCOPETAS scale [7]. A subsequent evidence-based evaluation conducted in Spain further demonstrated the utility of SCOPETAS for optimizing team composition during urgent interhospital transfers, confirming its applicability and safety in real-world clinical practice [8].

The initial validation of SCOPETAS showed excellent inter-rater reliability among physicians (κ = 1) and substantial agreement between actual clinical practice and scale recommendations (weighted κ = 0.68; *p* < 0.001) [7]. However, its application among nursing professionals has not been studied. Given the variability in pediatric IHT team composition and the absence of validated nursing-specific tools, assessing SCOPETAS validity in this professional group is crucial to ensure safe, efficient, and consistent care.

The primary objective of this study was to evaluate the validity of the SCOPETAS scale when applied by nursing professionals. Specifically, we aimed to determine inter-rater agreement with the physician gold standard and to analyze diagnostic performance and predictors of correct classification.

## 2. Materials and Methods

### 2.1. Design

This study used a cross-sectional validation design based on two standardized clinical vignettes previously employed in the original SCOPETAS physician validation study between December 2024 and February 2025. Nursing professionals independently applied the scale, and their decisions were compared with the established physician gold standard.

### 2.2. Participants

The study included nurses working in pediatric services across eight hospitals within a Spanish autonomous community: one tertiary hospital with comprehensive pediatric specialties, six hospitals with pediatric emergency and inpatient care, and one mixed adult–pediatric facility. All eligible nurses were informed about the study and invited to participate voluntarily. Those who agreed provided informed consent and accessed the online sociodemographic questionnaire. Participation was voluntary, anonymous, and non-incentivized. No exclusion criteria were applied.

### 2.3. Instrument

The SCOPETAS scale evaluates pediatric patient severity across three domains (neurological, cardiovascular, respiratory; 0–3 points each) and considers the presence of a significant diagnosis (Appendix A.1).

Total score (0–9) determines team composition:

• 0–1: Emergency Medical Technician (EMT)
• 2–4: Nurse + EMT
• ≥5: Physician + Nurse + EMT

A score of 3 in any domain or the presence of a significant diagnosis automatically indicates a full team. The Spanish version of the scale was previously validated among physicians using simulated scenarios, yielding a weighted κ = 0.685 (95% CI: 0.582–0.789; *p* < 0.001) [7].

### 2.4. Procedure

Following the methodology of the physician validation study, participants evaluated the same two standardized clinical cases (Appendix A.2 and Appendix A.3):

1. Severe case: Infant with bronchiolitis and severe respiratory distress requiring non-invasive ventilation—full team indicated (Physician + Nurse + EMT).
2. Mild case: Stable patient with uncomplicated acute appendicitis—EMT only indicated.

Participants received a printed packet containing the two standardized clinical vignettes, the SCOPETAS scale, and all necessary supporting materials. They completed the assessment on paper under unsupervised conditions to minimize external influence and ensure uniform case presentation. Subsequently, they entered their sociodemographic data and transcribed their responses into a Google Forms questionnaire. No time limit was imposed to allow careful application of the scale, mirroring real-world decision-making.

### 2.5. Variables

Collected data included demographic (age, sex), academic (highest degree, specialized training), and professional characteristics (years of experience, pediatric experience), as well as SCOPETAS-based transport team decisions for each vignette.

### 2.6. Data Analysis

Analyses were performed with SPSS v.21 and R v.4.3.3. Descriptive, bivariate, andmultivariate (logistic regression) analyses were conducted. ROC curves evaluateddiscrimination. Significance was set at *p* < 0.05.

Bayesian posterior distributions were estimated via Markov Chain Monte Carlo (100,000 iterations) under a minimally informative Beta-Binomial prior. For the analysis, library rstan from R v.4.3.3 was used, with 4 Marcov chains, 1000 warm-up iterations per chain, and a total number of 25,000 iterations per chain in 2 cores. For the convergence diagnostics, Gelman’s Rhat and the effective sample size were used as analytical tools, and graphically, the traceplot method was used to plot the time series of the posterior draws.

### 2.7. Ethical Considerations

The study was approved by the Regional Research Ethics Committee (No. 2024.466). Participation was voluntary and anonymous. No real patient data were collected, and all procedures complied with the Spanish Organic Law 3/2018 on Data Protection and Digital Rights.

## 3. Results

### 3.1. Participant Characteristics

Of the 175 nurses invited, 128 completed the assessment (response rate 73.1%). Participants were predominantly female (91%, n = 117) with a mean age of 39.5 ± 10.3 years. Most held a Bachelor’s/Diploma degree (64.1%), followed by Master’s (15.6%), Pediatric Nursing Specialist certification (19.5%), and Doctorate (0.8%). Mean professional experience was 15.8 years, including 6.2 years in pediatrics. Most participants worked in tertiary hospitals, primarily in pediatric emergency, inpatient, or intensive care units (Table 1).

### 3.2. Application of SCOPETAS

In Case 1, 91.4% correctly identified the full team requirement; in Case 2, 73.9% selected the EMT-only team. Full agreement across both cases occurred in 66.9% (95% CI: 58–75%). High respiratory scores predominated in Case 1, while Case 2 showed mostly zero values (Table 2).

### 3.3. Factors Associated with Accuracy

Longer pediatric experience slightly reduced accuracy (OR = 0.992; 95% CI: 0.984–0.999; *p* = 0.03). Holding a Master’s degree decreased the odds of correct classification in both cases and for Overall Agreement (OR = 0.27; 95% CI: 0.11–0.70; *p* = 0.006) (Table 3).

### 3.4. Diagnostic Performance

For Case 1, sensitivity was 64.7%, specificity 83.3%, PPV 97.4%, and NPV 19.6%.

For Case 2, sensitivity was 80.0%, specificity 48.5%, PPV 81.7%, and NPV 45.7%.

**Figure 1:**
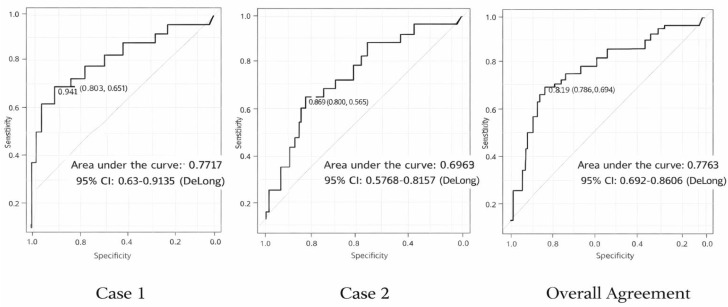
Receiver operating characteristic (ROC) curve for Case 1, Case 2 and Overall Agreement, showing the discriminative performance of nursing decisions compared with the physician gold standard.

Overall full-agreement model: sensitivity was 67.1%, specificity 81.0%, PPV 87.7%, and NPV 54.8%. Detailed indices are presented in Table 4 and Figure 1.

### 3.5. Comparison with Medical Decisions

Compared to physicians’ decisions (gold standard), nursing decisions achieved a sensitivity of 91%, specificity 74%, LR+ 3.48, LR-0.12, and substantial evidence weight (positive 5.4 deciban; negative −9.1 deciban) (Table 5).

## 4. Discussion

Pediatric Interhospital Transport (IHT) triage remains insufficiently explored in nursing research despite its growing relevance in increasingly complex care environments. This study provides the first validation of the SCOPETAS scale among nursing professionals, extending its previous physician-based validation to the nursing context and addressing a key gap in pediatric transport decision-making.

In this study, the application of the SCOPETAS scale by nursing professionals demonstrated high concordance with physicians’ decisions, particularly in the severe scenario. The lower item-level agreement observed in the respiratory domain of Case 1—where 56% of participants assigned a score of 2 despite the presence of BiPAP, which corresponds to a score of 3 according to the scale—indicates a discrepancy between domain-specific scoring and the final team selection. Importantly, SCOPETAS incorporates redundant decision pathways inherited from the original PT3 instrument, whereby a full-team recommendation is triggered either by a score of 3 in any single domain or by a cumulative total score ≥3 across domains. Consequently, even when the respiratory domain was underscored, participants frequently reached a total score consistent with the correct full-team assignment. This redundancy enhances the safety and robustness of the tool, helping to preserve final decision accuracy despite isolated item-level errors, while also underscoring the need to reinforce targeted training in domain-specific scoring—particularly regarding advanced respiratory support modalities—to ensure precision at both the item and overall decision levels.

The high positive predictive value indicates that when nurses recommend a full team, the decision is almost always appropriate, reinforcing the scale’s reliability and its potential to optimize specialized resources. The inverse association between holding a Master’s degree and accuracy should be interpreted cautiously. The small size of this subgroup, limited historical involvement of nurses in transport team decisions within the Spanish healthcare system, and heterogeneity in postgraduate training pathways may all contribute to this finding. Rather than suggesting a deficit, these results underscore the opportunity to strengthen structured, competency-based training in SCOPETAS and progressively integrate nursing professionals into transport-related decision-making. Interpretation of predictive values must consider the study design; because the two vignettes represent extreme severity levels, PPV and NPV reflect the imposed case mix rather than real epidemiological prevalence. These values should therefore not be extrapolated directly to clinical settings, where the proportion of severe cases varies across hospitals and time periods. The intermediate SCOPETAS category (Nurse + EMT) could not be evaluated because the study intentionally replicated the original physician validation, which included only mild and severe cases. This approach ensured methodological comparability but limited assessment of the scale’s full operational range. Future research incorporating intermediate-severity scenarios will be essential to evaluate the discriminative capacity and practical applicability of this category in nursing practice.

No significant associations were found with age, sex, or hospital type, suggesting broad applicability across diverse clinical settings. The high concordance with physicians (sensitivity 91%, specificity 74%) reinforces SCOPETAS as a robust tool to support nursing decision-making in pediatric IHT. Internationally, countries with advanced pediatric transport systems, such as the UK, have implemented standardized triage protocols, though these remain predominantly physician-led [9,10,11]. 

Validating SCOPETAS for nursing professionals represents an innovative contribution that may enhance nursing autonomy, patient safety, and equitable access to specialized pediatric care.

From an organizational perspective, integrating SCOPETAS could improve system efficiency by accurately identifying cases that do not require full teams, thereby optimizing resource allocation without compromising safety. Digital integration may further facilitate its clinical use and enable continuous quality monitoring. Nonetheless, multicenter studies with larger samples and a broader range of clinical scenarios will be necessary to rigorously assess the external validity and real-world applicability of SCOPETAS in nursing practice.

### Limitations

This study used simulated scenarios within a single autonomous community, limiting external validity. The online data collection format precluded replication of real-world transfer dynamics. Moreover, the intermediate configuration (Nurse + EMT) was not assessed, warranting future research.

## 5. Conclusions

The findings of this study suggest that SCOPETAS may contribute to improved patient safety and more efficient resource allocation when applied by nursing professionals. Although the tool demonstrated promising validity in this simulated context, additional research—ideally incorporating real-world clinical environments, a broader range of clinical scenarios, and multicenter designs—is needed to more accurately determine its practical applicability and to guide its potential integration into routine nursing practice.

## Figures and Tables

**Table 1:** Sociodemographic and professional characteristics of the sample.

Variable	N = 128	n (%)	Mean (SD)
Sex			
Male	11	(9)	
Female	117	(91)	
Age group			
21–30 years	30	(23.4)	
31–40 years	44	(34.4)	
41–50 years	35	(27.3)	
51–60 years	16	(12.5)	
>60 years	3	(2.3)	
Academic qualification			
Diploma/Bachelor’s degree	82	(64.1)	
Master’s degree	20	(15.6)	
Pediatric Nursing Specialist	25	(19.5)	
Doctorate	1	(0.8)	
Service/Department			
Pediatric Emergency	31	(24.2)	
Pediatric Inpatient Unit	30	(23.4)	
PICU	21	(16.4)	
Other	46	(35.9)	
Professional experience (years)			15.8 (9.86)
Pediatric experience (years)			6.2 (7.68)

**Table 2:** SCOPETAS scores in both cases.

SCOPETAS Score	0	1	2	3
Case 1				
Neurological	107 (84%)	18 (14%)	3 (2%)	0 (0%)
Cardiovascular	21 (16%)	92 (72%)	14 (11%)	1 (1%)
Respiratory	0 (0%)	8 (6%)	72 (56%)	48 (38%)
Case 2				
Neurological	127 (99%)	1 (1%)	0 (0%)	0 (0%)
Cardiovascular	115 (90%)	13 (10%)	0 (0%)	0 (0%)
Respiratory	126 (98%)	2 (2%)	0 (0%)	0 (0%)

**Table 3:** Factors associated with correct team selection.

Variable	Case 1 OR (95% CI) *p*-Value	Case 2 OR (95% CI) *p*-Value	Overall Accuracy OR (95% CI) *p*-Value
Intercept	3.08 (0.54–17.6) 0.206	3.59 (2.24–5.70) <0.001	2.35 (0.90–6.13) 0.080
Years of nursing experience	1.15 (0.97–1.37) 0.177	—	1.02 (0.96–1.07) 0.566
Years of pediatric experience	1.09 (0.86–1.37) 0.482	—	1.13 (0.96–1.33) 0.144
Interaction (nursing × pediatric experience)	0.992 (0.984–0.999) 0.0305	—	0.994 (0.988–1.000)
Master’s degree (yes)	0.224 (0.057–0.874) 0.0325	0.224 (0.057–0.883) 0.0371	0.271 (0.106–0.695) 0.0066

**Table 4:** Diagnostic estimates.

Estimate	Case 1 Value (95% CI)	Case 2 Value (95% CI)	Overall Accuracy (95% CI)
True prevalence	0.906 (0.842–0.951)	0.742 (0.657–0.815)	0.669 (0.580–0.750)
Sensitivity	0.647 (0.552–0.733)	0.800 (0.705–0.875)	0.671 (0.560–0.769)
Specificity	0.833 (0.516–0.979)	0.485 (0.308–0.665)	0.810 (0.659–0.914)
Positive predictive value	0.974 (0.909–0.997)	0.817 (0.724–0.890)	0.877 (0.772–0.945)
Negative predictive value	0.196 (0.098–0.331)	0.457 (0.288–0.634)	0.548 (0.417–0.675)
Diagnostic accuracy	0.664 (0.575–0.745)	0.719 (0.632–0.795)	0.717 (0.630–0.793)
Positive likelihood ratio	3.879 (1.087–13.845)	1.553 (1.099–2.195)	3.521 (1.854–6.684)
Negative likelihood ratio	0.424 (0.298–0.604)	0.412 (0.242–0.704)	0.401 (0.291–0.570)
Weight of evidence (+test) [decibans]	5.887 (0.362–11.413)	1.911 (0.041–3.414)	5.467 (2.681–8.250)
Weight of evidence (−test) [decibans]	−3.726 (−5.258 to −2.190)	−3.851 (−6.162 to −1.524)	−3.979 (−5.361 to −2.441)

**Table 5:** Comparison between nursing and medical decisions.

	Case 1	Case 2	Total
Positive response	116	33	149
Negative response	12	95	107
Total	128	128	256

## Data Availability

The data presented in this study are not publicly available due to institutional or confidentiality restrictions.

## Public Involvement Statement

No public involvement in any aspect of this research.

## Guidelines and Standards Statement

This manuscript was drafted against the STROBE (Strengthening the Reporting of Observational Studies in Epidemiology).

## Abbreviations

The following abbreviations are used in this manuscript:

Abbreviation	Full Term
CI	Confidence Interval
EMT	Emergency Medical Technician
IHT	Interhospital Transport
κ	Cohen’s kappa coefficient
LR+	Positive Likelihood Ratio
LR–	Negative Likelihood Ratio
NPV	Negative Predictive Value
OR	Odds Ratio
PEWS	Pediatric Early Warning Score
PICU	Pediatric Intensive Care Unit
PPV	Positive Predictive Value
PT3	Pediatric Transport Triage Tool
ROC	Receiver Operating Characteristic
SCOPETAS	Spanish Cultural Adaptation of the Pediatric Transport Triage Tool

## Appendix A

### Appendix A.1

**Table A1:** **SCOPETAS scale**.

System	Score 0	Score 1	Score 2	Score 3
Neurological	Alert, playful, interactive; Glasgow 15; Within baseline status	Drowsy but responsive to stimuli; Consolable crying; Glasgow 13–14	Irritable, inconsolable; Hypotonic; Neurological focal signs; Glasgow < 12 or >2 points below baseline	Lethargic, confused; Loss of cough and gag reflex; Sedated or paralyzed
Cardiovascular	Pink; Capillary refill 1–2 s; HR and/or BP < 10% above or below normal values	Pale; Capillary refill 3 s; HR and/or BP ± 10% from normal	Mottled skin; Capillary refill 3–4 s; HR and/or BP ± 20%; Unrepaired duct-dependent congenital heart disease	Gray; Capillary refill < 1 s or >4 s; HR or BP ± 30%; Need for inotropes; Unstable arrhythmia
Respiratory	Normal respiratory rate; No respiratory effort	RR > 10 breaths/min above normal; Accessory muscle use; Nasal flaring; Agitated stridor; O_2_ < 3 L/min	RR > 20 breaths/min above normal; Resting stridor; O_2_ > 3 L/min; HFNC/CPAP; Continuous nebulization; Baseline tracheostomy or ventilator dependence; Respiratory muscle fatigue with altered baseline	RR > 5 breaths/min below normal; Hypoventilation with altered consciousness; Grunting with respiratory compromise; BiPAP; Invasive mechanical ventilation

**Significant Diagnoses**

**Airway**

- Pneumothorax with respiratory compromise
- Upper airway obstruction
- History of difficult airway with respiratory compromise
- Unstable mandibular fracture with potential airway compromise

**Neurological**

- Ventriculoperitoneal shunt dysfunction
- Newly diagnosed intracranial mass
- Intracranial hemorrhage
- Stroke
- Acute spinal cord disease or injury
- Status epilepticus or recurrent seizures difficult to control

**Gastrointestinal**

- Esophageal foreign body
- Volvulus/malrotation
- Perforated appendix
- Incarcerated hernia

**Surgical, Traumatic, and Burns**

- Burns > 5% TBSA on face/neck
- Burns > 10% TBSA
- Circumferential burns
- Orbital fracture with extraocular muscle involvement
- Severe splenic/hepatic laceration
- Compartment syndrome
- Neurovascular compromise
- Ovarian/testicular torsion < 6 h
- Digital or limb amputation
- Inhalation injury
- Polytrauma
- Penetrating injuries to head, chest, or abdomen
- Pelvic fracture

**Other**

- Suspected infection in immunocompromised patient with systemic involvement
- Acute vision loss
- Retrobulbar hematoma
- Diabetic ketoacidosis with altered mental status
- Potassium ≥ 6.5 mmol/L
- pH < 7.2
- Hemoglobin ≤ 5 g/dL with hemodynamic compromise
- Platelet count ≤ 20,000/μL with active bleeding

**Recommended Transport Team Based on Scoring**

**Maximum** **Score in a** **Single Category**

3 points: Physician + Nurse + EMT. Fastest transport possible.
2 points: Physician + Nurse + EMT
1 point: Nurse + EMT
0 points: EMT only

**Total score**

≥3 points: Physician + Nurse + EMT ± Fastest transport possible
1–2 points: Nurse + EMT
0 points: EMT only

**Presence of significant diagnosis**

Yes: Physician + Nurse + EMT ± Fastest transport possible

### Appendix A.2. Case 1

Personal History:

A 23-month-old patient, approximately 10 kg in weight. Immunizations up to date. No known drug allergies. No previous episodes of dyspnea.

Family History:

Father with asthma.

Current History:

The patient presented with a 12 h history of dyspnea. He had been assessed 24 h earlier at his primary healthcare center and diagnosed with mild bronchiolitis (no home treatment prescribed). The child was brought by his mother to the emergency department of a secondary-level hospital. On arrival, clinical examination revealed intercostal, subcostal, and supraclavicular retractions, a respiratory rate of 70 breaths/min, oxygen saturation (room air) of 87%, temperature of 39.9 °C, and generalized hypoventilation. Nebulized salbutamol 2.5 mg (two doses) was administered, intravenous access was established, and methylprednisolone 2 mg/kg and paracetamol 15 mg/kg were given.

The patient remained on reservoir-mask oxygen therapy. Following intervention, respiratory effort improved, although three-level retractions persisted. Air entry improved bilaterally, with occasional inspiratory wheezes, respiratory rate of 55 breaths/min, and oxygen saturation on room air 89% (rising to 99% with reservoir mask).

Given the clinical picture, non-invasive mechanical ventilation with bilevel positive airway pressure (BiPAP) was initiated, and transfer to a tertiary care center was arranged.

Clinical Status at the Time of Transfer:

Respiratory rate 55 breaths/min; oxygen saturation (BiPAP, FiO_2_ 45%) 95–96%; heart rate 189 bpm; blood pressure difficult to obtain but skin pale (constitutional) with good capillary refill (<3 s). Persistent three-level retractions. Alert and responsive.

### Appendix A.3. Case 2

Personal History:

An 8-year-old patient, approximately 30 kg in weight. Immunizations up to date. No known drug allergies. No prior surgical history.

Family History:

Non-contributory.

Current History:

Abdominal pain for approximately 24 h, continuous in nature, initially periumbilical and later localized to the right iliac fossa, without documented fever. Associated nausea and loss of appetite. The patient presented to the emergency department of a secondary-level hospital for evaluation. On arrival, physical examination revealed positive Blumberg and psoas signs. Abdominal ultrasound and blood tests were performed. The ultrasound showed findings compatible with uncomplicated acute appendicitis, and laboratory results were unremarkable. Given the clinical findings, the pediatric surgery department at the regional referral hospital was contacted, and transfer was arranged for surgical evaluation.

Clinical Status at the Time of Transfer:

Eupneic, afebrile, blood pressure 90/70 mmHg, heart rate 95 bpm, adequate capillary refill, conscious, oriented, and cooperative. Pain score (VAS) = 2.

Devices: Peripheral intravenous line in right antecubital fossa, flushed with saline.
